# Elbow Joint Position and Force Senses in Young and Adult Untrained People and Gymnasts

**DOI:** 10.3390/ijerph19137592

**Published:** 2022-06-21

**Authors:** Bartłomiej Niespodziński, Jan Mieszkowski, Stanisław Sawczyn, Kazimierz Kochanowicz, Adam Szulc, Mariusz Zasada, Andrzej Kochanowicz

**Affiliations:** 1 Department of Biological Foundations of Physical Education, Institute of Physical Education, Kazimierz Wielki University, Sportowa 2, 85-091 Bydgoszcz, Poland; aszul@ukw.edu.pl (A.S.); marzas@ukw.edu.pl (M.Z.); 2 Department of Gymnastics and Dance, Gdansk University of Physical Education and Sport, 80-336 Gdansk, Poland; mieszkowskijan@gmail.com (J.M.); stanislaw.sawczyn@awf.gda.pl (S.S.); andrzej.kochanowicz@awf.gda.pl (A.K.); 3 Department of Theory of Sport, Gdansk University of Physical Education and Sport, 80-336 Gdansk, Poland; kazimierz.kochanowicz@awf.gda.pl

**Keywords:** sense of force, proprioception, training, artistic gymnastics

## Abstract

Joint position (JPS) and force senses (FS) are the proprioception modalities. While the development of JPS was investigated both in children/adult and athlete/untrained conditions, there is a lack of insight into the development of FS. Overall, 28 gymnasts and 25 untrained controls underwent proprioception testing. They were divided into two groups: 9 to 11-year-old boys (13 gymnasts and 10 non-athletes) and 18 to 25-year-old adults (15 gymnasts and 15 non-athletes). The testing was performed at an isokinetic dynamometer and included elbow JPS and FS (20% and 50% maximal voluntary contraction) tasks. Children had two times higher error in JPS (*p* < 0.01) and 50% higher errors in FS of both flexor (*p* < 0.001) and extensor muscles (*p* < 0.05) in comparison with adults. Only in the 50% maximal voluntary contraction task, gymnasts showed 33% lower error than the controls (*p* < 0.01). Untrained boys presented 54%, 132%, and 169% higher error for elbow flexor performance than young gymnasts, untrained adults, and adult gymnasts, respectively (*p* < 0.01). The 9 to 11-year-old participants were characterized by a lower precision of JPS and FS performance in comparison with adults. Gymnastic training can possibly accelerate the development of FS when higher loads are considered.

## 1. Introduction

Proprioception is a fundamental sense of the human body. It allows individuals to move their bodies in space, and move their body parts in relation to one another. Moreover, proprioception plays a key role in joint injury prevention [1], especially after such incidents as anterior cruciate ligament ruptures [2]. Proprioception is mainly associated with the ability to detect passive motion, joint position sense (JPS), and other senses including balance, heaviness, effort, force or tension [3]. While the attention of researchers has been mostly given to kinesthesia, which is related to joint position and movement [4], less focus has been set on force sense (FS), which also influences joint movement [3]. Force sense gives the ability to distinguish or match different levels of torque produced by a muscle over a joint and allows one to adjust the force output, thus minimizing the energy expenditure during various tasks. Force sense, as described by Proske and Allen [5], incorporates modalities such as sense of heaviness, effort, and tension, which depend on task. Force sense is driven by both the peripheral factors, such as tendon organs, cutaneous receptors, muscle spindle activity, and the central nervous system, probably in the form of corollary discharges or an efferent copy of the descending motor command [6]. The dominance of one or the other system and interaction between them are modality-dependent and are still to be revealed [5].

Research concerning the development of proprioception has focused mainly on aspects such as balance [7] or kinesthesia [8,9]. It was shown that during childhood [8] as well as adolescence [10], JPS performance increases with age. As the peripheral component is pronounced in JPS, those changes are related mostly to the myelination process in maturating afferent nerves [11]. Similar to kinesthesia, it has been reported that other somatosensations (touch, haptic ability) increase with age from 2.5 to 18 years. On the other hand, there is a lack of insight regarding the development of FS, which also uses, at least in part, the afferents from muscle spindles [12].

It is maintained that proficiency in the kinesthetic aspects of proprioception underlies high competition levels in sports [2]. Athletes of different sports [13,14] or professional dancers [15], throughout many years of training, achieve better kinesthetic performance in comparison with untrained people. The reason for the increased proprioceptive performance in athletes could be the selection of individuals with inherent predispositions or training-induced enhancement in motor control. These can include peripheral changes, for example increased sensitivity of muscle and tendon receptors, as well as central changes in processing and facilitation [1]. Children are especially prone to training stimuli that accelerate their motor control development to the level observed in adults [16].

Besides kinesthesia, the ability to perfectly control muscle contraction is required to learn and master the complex technique necessary for elite athletes to achieve top level performance, especially in sports characterized by multi-joint and multidimensional maneuvers of the body. Examples of such sports are gymnastics, dancing, or diving. While the impact of training on balance development [17] or on kinesthetic aspects of proprioception development, e.g., JPS or detecting movement threshold [1,2,18], has been investigated to some extent, the insight into training influence on FS development is still limited. It was previously shown that long-term sports training had an effect on FS [14]; however, most of the studies investigated only one time point of the sports career, i.e., the mature period in athletes. Thus, the influence of sports training on developmental changes in proprioception (especially FS) is yet to be revealed.

The aim of the study was twofold: (1) to show the differences in JPS and FS in elbow joint of boys and adults; and (2) to evaluate the impact of long-term gymnastic training on the analyzed senses in young people and adults.

## 2. Materials and Methods

### 2.1. Experimental Approach to the Problem

An investigation based on an observational cross-sectional study design was used to evaluate the level of proprioception in terms of JPS and FS in children and adults. Participants belonging to the gymnasts or control group and to a particular age group acted as independent variables, while the proprioception outcome constituted dependent variables.

### 2.2. Participants

A total of 53 males took part in the study. The study group consisted of 28 gymnasts, divided into two age groups: 9 to 11-year-old boys (G1, *n* = 13) and 18 to 25-year-old young adults (G2, *n* = 15). The control group involved age-matched untrained children (C1, *n* = 10) and young adults (C2, *n* = 15). The characteristics of particular groups are presented in Table 1.

All gymnasts had started their gymnastic training as 6 to 7-year-olds, and were elite athletes or at least prospective gymnasts at the current training stage. The young gymnasts were at the basic stage of training, while the adult gymnasts were at a special stage of training. All the measurements took place in the preparatory training period. The gymnastic training mainly consisted of explosive and strength endurance exercises with body weight load (jumping, swings on apparatuses, etc.), as well as coordination training (balance on apparatuses, body maneuvers in the air, etc.). The control group consisted of participants who performed unstructured leisure physical activity no more than three times a week. The untrained children also attended obligatory physical education classes 3 × 45 min per week. The participants aged 9 to 11 years were at Tanner stage 1 or 2 of maturation. The study was performed in accordance with the Declaration of Helsinki and approved by the Bioethics Committee at the Regional Medical Chamber in Ethics Committee at the Regional Medical Chamber in Gdańsk (approval number: KG-12/15). All participants or their legal guardians provided their informed consent to participate in the study.

### 2.3. Procedures

For each participant, two proprioception tests were made: reproduction of joint angle for JPS and reproduction of force for FS. Both tests were performed unilaterally as bilateral matching tests are believed to make it difficult to distinguish between peripheral and central components of the tested proprioception performance [12]. The JPS and FS performance were tested in a random order. Before proper testing, each participant underwent a familiarization session with both procedures using random and different than testing target values.

#### 2.3.1. Joint Position Sense

The JPS test was comprised of memorized ipsilateral tasks where both the passive and active reproduction of the target elbow angle were evaluated in a random order. The task was performed on a Biodex System 4 Pro dynamometer (Biodex Medical Systems, Inc. Shirley, NY, USA). The participants were sitting in an adjustable seat with their elbow placed on a leather pad. The shaft of the dynamometer’s arm was aligned with the physiological rotation axis of the elbow in the sagittal plane. The glenohumeral joint was flexed in 45°; the forearm and hand holding the arm of the dynamometer in the investigated limb were set in neutral position. The position was stabilized with leather straps. In both types of tasks, the target angle was 90° of elbow flexion and before each trial; the participant’s elbow joint was passively set to the target angle to allow position memorization for 3 s. Next, the elbow joint was randomly extended in range of 15 to 60° to the position from where the particular trial would start. In passive reproduction mode, the participant’s elbow was moved passively by the device at a constant motion of 0.5°·s^−1^. During active reproduction mode, participants’ actively flexed their elbow, stopping at the point where they felt they had reached target position. In both reproduction modes, participants indicate target position by pressing a button held in the contralateral hand. The indicated angular positions were used for further analysis. For each task, three trials were performed. During the whole task, the participants were blindfolded and wore headphones to eliminate any additional cues. Each time, before the memorizing part or actual testing, the participants performed isometric co-contraction of elbow flexors and extensors to unify the conditioning effect of muscle contraction on the thixotropy effect [19].

#### 2.3.2. Force Sense

The ability to distinguish muscle force elicited by participants was evaluated with a memorized ipsilateral force reproduction task. The equipment and the position during the force reproduction task were the same as for the JPS test with 90° elbow flexion. Before testing, the participants performed three isometric 5 s maximal voluntary contractions (MVC). The result with the highest torque was used as the reference value for the force reproduction task. The subjects performed MVCs for both flexion and extension. Each attempt was followed by at least 1 min of break, and the actual force reproduction task started 10 min after MVC testing completion. During FS testing, it is crucial to provide participants with appropriate instructions that will allow our study to distinguish which FS modality is tested: sense of force, heaviness, or effort [5]. In this study, the subjects were asked to focus on the force (tension) produced over the joint, not the effort to achieve the target force level. Thus, the tested outcome should be mainly derived from the sense of force (muscle spindles and Golgi tendon organs) and tactile stimuli from the dynamometer handle. During this task, the participant, with the help of online visual feedback, had 3 s to memorize the target level of force. Then, after obligatory muscle relaxation after the next 5 s, the subjects were asked to reproduce the target force level and maintain it for 5 s. It was previously shown that difference between adult gymnasts and untrained peers in FS performance can be associated with level of target force [14]. Thus, the force reproduction included reproduction of 20% and 50% of MVC, evaluated in a random order. For each force level, three attempts were recorded. During the force reproduction, the participants were blindfolded and wore headphones to eliminate any additional cues. When feeling that they had achieved the target force level, they gave a verbal signal to register the attempt. The average value taken from the middle 3 s of the registered MVC-normalized torque value of each attempt was used in further analysis.

### 2.4. Data Analysis

The following JPS and FS outcomes were analyzed: absolute error (AE) and constant error (CE). Absolute error was calculated as a mean absolute difference between the target value and the test value of three trials, while CE was determined as a mean difference of the trials. Absolute error was used to estimate the overall acuity (magnitude of error) of JPS and FS, whereas CE showed the direction of the error. The JPS outcome was analyzed in degrees. As force reproduction was performed relative to MVC, the FS outcome was analyzed in percentage points. The reliability of JPS and FS tests on the Biodex System was previously established to be sufficient, with the intra-class correlation coefficient of 0.78 and 0.81, respectively [20].

### 2.5. Statistical Analyses

To assess the difference between particular age groups and the impact of gymnastic training, a set of two-way (2 × 2) ANOVA tests were performed. The first factor, age, indicated child-adult differences, represented by 9 to 11-year-old and 18 to 25-year-old participants. The second factor, group, indicated the impact of gymnastic training, and divided the participants into two groups: gymnasts and non-athletes. The assumptions of the ANOVA tests, including normal distribution (Shapiro-Wilk test) and homogeneity of variance (Levene test), were checked before, and showed no basis to reject them. In the case of a significant effect of the factors interaction, Tukey’s post-hoc tests were performed. All results were shown as mean ± standard deviation. The effect size was estimated by eta-squared statistics (ƞ^2^), where values equal or less than 0.01, 0.06, 0.14 and more than 0.14 indicated trivial, small, moderate, and large effects, respectively [21]. The level of significance for all test was set at α = 0.05. The required sample size of 52 participants was estimated with G*Power software ver. 3.1.9.4. (Franz Faul et al., Universität Kiel, Kiel, Germany) for a large effect size, and a power of 0.80. All tests were performed with the Statistica 13.3 software (TIBCO Software Inc., Palo Alto, CA, USA).

## 3. Results

### 3.1. Joint Position Sense

**Figure 1:**
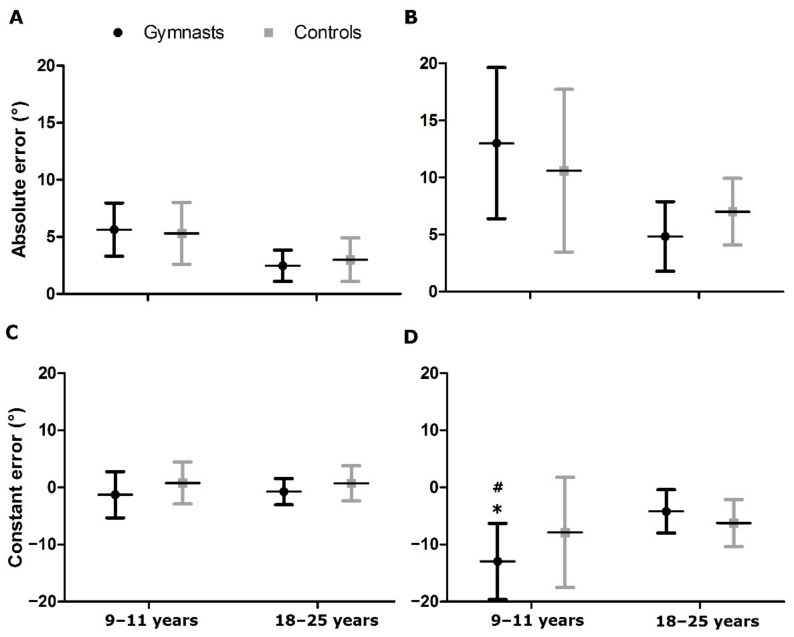
Ipsilateral joint position sense testing in elbow joint during active reproduction (**A**,**C**) and passive reproduction (**B**,**D**). Significant difference at *p* < 0.01 with * 9–11-year-old controls and # 18–25-year-old gymnasts.

The JPS results are presented in Figure 1. Both active (F = 22.53; *p* = 0.0001; ƞ^2^ = 0.32) and passive (F = 17.59; *p* = 0.0011; ƞ^2^ = 0.27) reproduction in terms of AE showed a significant effect of age. Children had two times higher AE in comparison with adults. Similarly, CE of passive reproduction revealed the effect of age factor in form of about two times higher error in children (F = 9.1; *p* = 0.0039; ƞ^2^ = 0.16). Neither in AE nor in CE of active and passive reproduction was the effect of group factor seen. However, the CE in passive reproduction showed significant interaction (F = 4.33; *p* = 0.04; ƞ^2^ = 0.08). The 9 to 11-year-old gymnasts presented three and two times higher errors directed below the target value in comparison with 18 to 25-year-old gymnasts, and the control group of the same age, respectively (Figure 1D).

### 3.2. Force Sense

**Figure 2:**
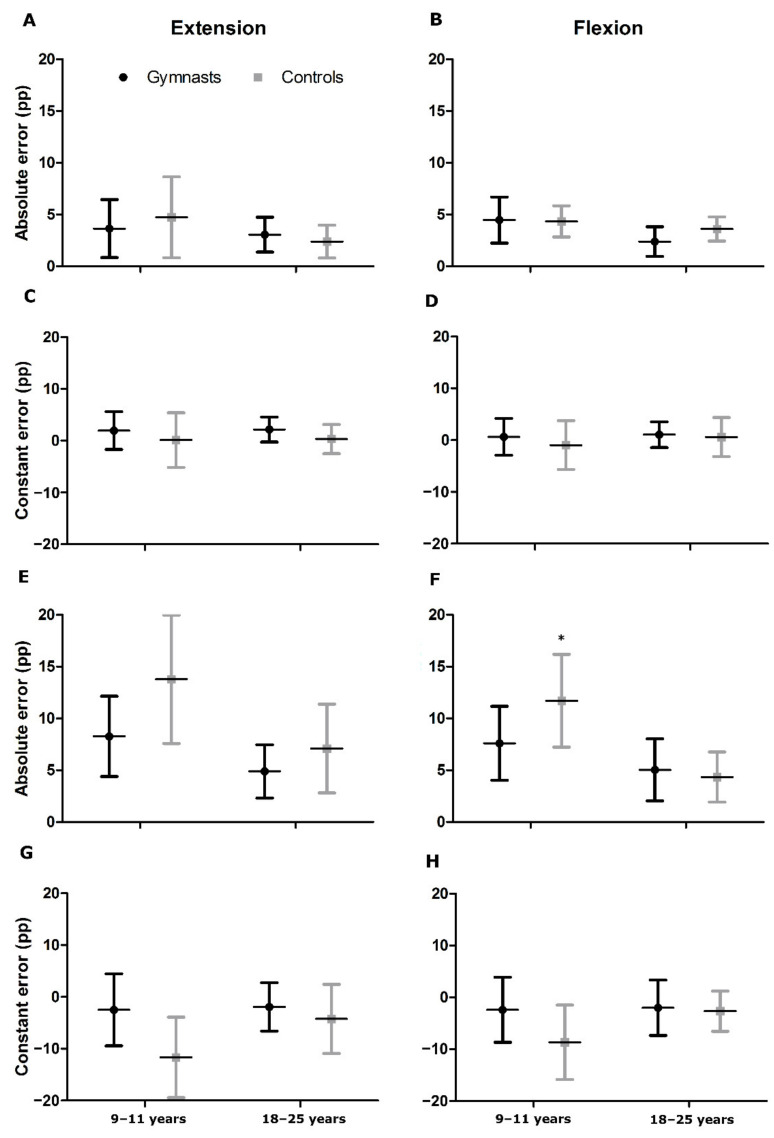
Ipsilateral force sense results in elbow joint for 20% (**A**–**D**) and 50% (**E**–**H**) of maximal voluntary contraction. Pp—percentage point, * significant difference with the remaining groups at *p* < 0.01.

The FS results are presented in Figure 2. The results of the ANOVA tests are depicted in Table 2. With reference to the 20% MVC force reproduction task, the outcome for extensors and flexors were similar. The effect of age was seen only in AE, where children presented about 50% higher mismatch than adults, regardless of sports training. While there was no effect of age or interaction in CE, gymnasts showed a tendency to overshoot the target force during extension, while in the control group, the error was evenly distributed.

In the case of the 50% of MVC force reproduction task, both age and group effects were visible in AE for elbow extension. The difference between children and adults rose up to 78%, and the gymnasts showed a 33% lower error in comparison with the control group. Similarly, in CE, both analyzed factors had an effect. All participants undershot the target force, yet children mismatched two times more than adults. The gymnasts also showed a 70% lower value of CE. While there was no significant interaction between the two factors, a tendency was observed where the untrained boys surpassed the rest of the analyzed groups (Figure 2G). The same results, regarding the significance of the two main effects and tendency of interaction in CE, were observed for the 50% of MVC elbow flexion force reproduction task.

The results of the 50% of MVC force reproduction task for flexors in AE were similar to those obtained in extensors; however, in terms of group effect, only the tendency of 14% lower value of error in gymnasts in comparison with the control group was revealed. Conversely, the interaction between both analyzed factors proved significant. The untrained boys presented 54%, 132%, and 169% higher AE than young gymnasts, untrained adults, and adult gymnasts, respectively (Figure 2F).

## 4. Discussion

One of the study aims was to show the differences between boys and adults in FS and JPS. Higher AE values were observed in children in comparison with adults for both FS and JPS. The results of JPS are in line with the kinesthetic research by other authors [8,9,10]. It was reported [8] that adolescents (16 to 18-year-olds) had a 50% reduction in matching error in comparison with 8 to 10-year-old children for each tested modality, i.e., ipsi- and contralateral remembered and bilateral matching. It was also indicated [8] that the main reason for increased proprioception should be the improvement of central sensorimotor processes, including increased myelination of fiber tracts [11] and amelioration of the synaptic connections based on experience [22]. On the other hand, Duzgun, et al. [23] showed that maturation measured by the Tanner stage scale had no effect on JPS in the ankle joint. However, it should be noted that the authors [23] observed 20– to 30% lower matching errors in late-stage adolescents in comparison with early-stage ones, although the results did not reach significance. Thus, the different results in the present study could be due to methodological aspects.

Besides the differences in JPS, the results also presented FS improvement from childhood to adult age. The reduced FS reproduction errors in adults were observed for both 20% and 50% MVC tasks. To the authors’ knowledge, this is probably the first time where FS differences have been shown between children and adults. While there is no clear linear correlation between JPS and FS [14], some common mechanisms could be partially utilized by both modalities of proprioception. One of them could be the sense of heaviness, which plays a role in FS but also gives additional input due to gravity forces during active reproduction tasks in vertical JPS tests [24]. The difference between a child and an adult in FS acuity could be in some part similar to that in JPS, if one considers the myelination of the fiber tracks or amelioration of the synaptic connections, but it also could be explained by the increased type II motor-unit requirement in adults, especially in 50% MVC force reproduction [25]. It is possible that the additional motor-unit pool during the contraction increases the sensitivity of muscle spindles through α-γ motor neurons co-activation [26]. It should be noted that while the instructions for participants were provided to focus on the sense of force, possibly other modalities were also utilized to some extent during force reproduction, such as sense of effort or heaviness—especially that both JPS and FS tasks were performed in the vertical plane, which allows the gravity force to influence the performance. However, this mainly applies to the active reproduction of JPS, as in other conditions the upper limb lay supported on leather pads. The vertical plane of elbow movement was chosen because it is more natural in daily life activities and more typical of gymnastic exercises.

The second aim was to evaluate the impact of gymnastic training on the development of proprioception. The training-induced enhancement of JPS can be associated with the increased neuromuscular control, including, among others, muscle coordination and tonus regulation [27]. Some authors showed that just eight weeks of strength training without additional proprioceptive components was able to increase JPS performance [18]. It was suggested [18] that an important factor in training-induced enhancement of JPS was the appropriate stimulation of the γ motor neuron of muscle spindles by similar loads applied in agonist and antagonist muscles. This improves the force balance around the joint and thus increases the muscle spindle sensitivity via antagonistic muscle balance in the α-γ motor neuron co-activation [26]. Previous works [2] showed that the competition level in sports was associated with proprioceptive skills, such as differentiating small differences between active movements. Likewise, expertise in taekwondo training was proved to be related with better knee JPS performance among 11 to 14-year-old adolescents [28]. Finally, a study by Aydin, et al. [29] reported that adolescent female gymnasts aged 10 to 17 years exhibited better proprioception in terms of ankle kinesthesia (detecting motion threshold and JPS) in comparison with their untrained counterparts. A similar outcome was previously observed in knee and elbow joints among adult gymnasts [14,30]. In the present study, although the adult gymnasts exhibited lower AE of the elbow joint, the outcome did not reach significance and there was no difference among 9 to 11-year-old participants. One of the reasons for the lack of JPS difference in young gymnasts could be due to the earlier introduced lack of balance between the agonist and antagonist muscles. Gymnasts improved their neuromuscular control through long-term training; however, if one considers the elbow joint, the most focus is directed to elbow extensors, which serve as a support for the bodyweight during most gymnastic exercises [31]. The difference between the presented studies could be joint- and/or sex-related, as gymnasts’ better kinesthetic performance was observed for lower limb and among females [32]. This could be associated with the fact that gymnasts utilize their lower limb joints to maintain balance constantly during all floor routines, as well as during landings in vaults and dismounts from apparatuses. In addition, the artistic gymnastics compromised typical balance apparatus (balance beam), solely designed for female athletes. In the light of the previous studies, these results suggest that in young gymnasts, it is likely that more gymnastic training experience would lead to higher performance in JPS. However, future research including different joints and kinesthetic modalities should be carried out to verify that.

What is interesting, young gymnasts’ passive reproduction in JPS was characterized by a larger undershoot of target angle than that of their untrained counterparts. It could be associated with the habitual position where the elbow joints are held and locked in full extension, which helps young gymnasts to support their bodyweight in many gymnastic routines, such as handstands and body swings, until they develop enough muscle strength to control the elbow joint. This could also result from the earlier mentioned higher muscle strength of elbow extensor muscles.

In contrast, in the current study, the FS results showed that the overall gymnasts’ performance was better than that in controls when the target force level was higher. The novel outcome of this study revealed that the young gymnasts made fewer 50% MVC force reproduction errors in comparison with the untrained counterparts. The reason might be the aforementioned lower type II motor-unit requirement in children [25]. The young gymnasts presented increased capability in terms of rate of force development [16,33], which in large portion depends on the type of motor units, which suggests that gymnastic training could have enhanced their neuromuscular properties similar to those represented by adults. Therefore, the increased utilization of type II motor units could explain in part why the gymnasts showed higher performance in comparison with non-athletes only in the 50% MVC force reproduction task, as in the higher target force (50% vs. 20% of MVC), the representation of fast and precise motor units would be greater [34]. While the differences in FS were significant only in the case of elbow flexors, a similar tendency was noted for elbow extensors. It was previously shown that a force reproduction task with target force level above 20% of MVC was muscle group-dependent [14], which could explain the observed differences between elbow flexors and extensors. What is more, as mentioned earlier, long-term gymnastic training is directed toward muscle strength of elbow extensors [31], which would be consistent with the observed negative correlation between muscle strength of the antagonists and FS performance [14].

It is hard to confront the findings as there are no studies focusing on the impact of training on the development of FS, especially in children. The previous study investigating the effects of gymnastic training in adults showed that gymnasts made lower errors in 50% MVC reproduction of FS tests; however, the difference did not reach significance [14]. One of the other studies [35] reported that six-weeks of strength training had no effect on FS in ankle joint, which stands opposite to the presented results. However, the authors [35] themselves pointed that their FS measures did not have statistical power and thus should be interpreted with caution. Moreover, the lack of effect could be due to the fact that the intervention was performed in participants with functional ankle instability and/or to the training itself, which was focused on muscle strength. In the present study, the subjects had no visible or reported joint instability and the gymnastic training, besides muscle strength, was focused on balance, among others. Lastly, in the present study, differences were observed only in the 50% MVC task, while in the study mentioned before [35], the test utilized only 20% and 30% of MVC, which, similarly to the current 20% MVC outcome, could be not enough to show the training effects.

It could be argued if the observed differences are related to the specific selection of children with a predisposition instead of gymnastic training. While the process of selection is included in gymnastics, the focus is set on overall physical fitness and the anthropometric characteristics that could be preserved in later years and adulthood. However, it cannot be excluded that these children also had higher proprioception capabilities, even though they had never been tested before in this matter. It was shown that proprioception in terms of JPS and FS was to a substantial degree genetically conditioned, with heritability indices ranging from 0.60 to 0.77 [20]. It was also pointed out [20] that the role of environmental factors such as training should not be ignored, although genetics are responsible for the maximal capabilities of individuals. Other studies confirmed that the proprioception modalities, e.g., JPS, could be affected by physical activity in young and old adults [36,37] by neuromuscular training [18], or even by an eight-week warm-up program [38]. Moreover, children and adolescents aged 6 to 15 years showed that they could improve their balance skills within 5 to 10 weeks of proprioception-oriented training [39,40]. Thus, the observed higher proprioception performance in gymnasts should be at least to some extent related to the gymnastic training.

## 5. Conclusions

The differences in proprioception in terms of JPS and FS were observed between boys and adults. The children and adolescents aged 9 to 11 years were characterized by lower precision of JPS and FS in comparison with adults. Gymnastic training can possibly accelerate FS development when higher loads are considered.

## Figures and Tables

**Table 1:** Characteristics of the participants.

Characteristics	9–11-Year-Olds		18–25-Year-Olds	
	Gymnasts	Controls	Gymnasts	Controls
Age (years)	11.07 ± 0.65	10.47 ± 0.73	20.20± 2.40	20.07 ± 0.80
Body mass (kg)	35.22 ± 4.98	37.54 ± 6.29	67.24 ± 5.05 *	73.17 ± 6.29
Body height (cm)	142.12 ± 6.69	144.5 ± 6.92	169.97 ± 3.87 **	178.26 ± 4.22
Training experience (years) ^a^	5.07 ± 0.65	-	14.2 ± 2.40	-
Training volume (hours/week) ^a^	14	-	24	-

^a^ Training experience and volume refers only to the structured gymnastic training. Significant difference with the control group at * *p* < 0.01, ** *p* < 0.001.

**Table 2:** Two-way analysis of variance test results in force reproduction tasks.

Task	Examined Muscles	Measured Error	Effect	F Statistics (df)	*p*-Value	Effect Size (ƞ^2^)	Significant Outcome
20% of MVC	Ext.	AE	Age	4.40 (1, 49)	0.04 *	0.08	A1 > A2
			Group	0.10 (1, 49)	0.75	<0.01	
			Group × age	1.60 (1, 49)	0.21	0.03	
		CE	Age	0.04 (1, 49)	0.84	<0.01	
			Group	3.50 (1, 49)	0.07	0.07	
			Group × age	<0.01 (1, 49)	1.00	<0.01	
	Flex.	AE	Age	9.81 (1, 49)	<0.01 **	0.17	A1 > A2
			Group	1.47 (1, 49)	0.23	0.03	
			Group × age	2.23 (1, 49)	0.14	0.04	
		CE	Age	0.96 (1, 49)	0.33	0.02	
			Group	1.04 (1, 49)	0.31	0.02	
			Group × age	0.32 (1, 49)	0.57	<0.01	
50% of MVC	Ext.	AE	Age	18.25 (1, 49)	<0.01 ***	0.27	A1 > A2
			Group	10.74 (1, 49)	<0.01 **	0.18	G < C
			Group × age	1.97 (1, 49)	0.17	0.04	
		CE	Age	4.91 (1, 49)	<0.03 *	0.09	A1 < A2
			Group	10.14 (1, 49)	<0.01 **	0.17	C < G
			Group × age	3.65 (1, 49)	0.06	0.07	
	Flex.	AE	Age	28.70 (1, 49)	<0.01 ***	0.37	A1 > A2
			Group	3.42 (1, 49)	0.07	0.07	
			Group × age	6.69 (1, 49)	0.01	0.12	G1, G2, C2 < C1
		CE	Age	4.17 (1, 49)	<0.05 *	0.08	A1 < A2
			Group	4.87 (1, 49)	0.03 *	0.09	C < G
			Group × age	3.19 (1, 49)	0.08	0.06	

AE—absolute error, CE—constant error, df—degree of freedom, Ext.—extensors, Flex.—flexors, MVC—maximal voluntary contraction, A—age group, C—control group, G—gymnasts, (X)1—9–11-year-olds, (X)2—18–25-year-olds, significant difference at * *p* < 0.05, ** *p* < 0.01, *** *p* < 0.001.

## Data Availability

Available upon request.
